# Supplementary material for: Dissecting the Functional Organization of the C. elegans Serotonergic System at Whole-Brain Scale
Source: bioRxiv. 2023 Jan 18:2023.01.15.524132. Preprint. [Version 1] doi: 10.1101/2023.01.15.524132 (PMC9882198; doi:10.1101/2023.01.15.524132)
Supplement: 1 [file NIHPP2023.01.15.524132v1-supplement-1.pdf]

bioRxiv preprint doi: https://doi.org/10.1101/2023.01.15.524132; this version posted January 18, 2023. The copyright holder for this preprint (which was not certified by peer review) is the author/funder, who has granted bioRxiv a license to display the preprint in perpetuity. It is made available under aCC-BY 4.0 International license.

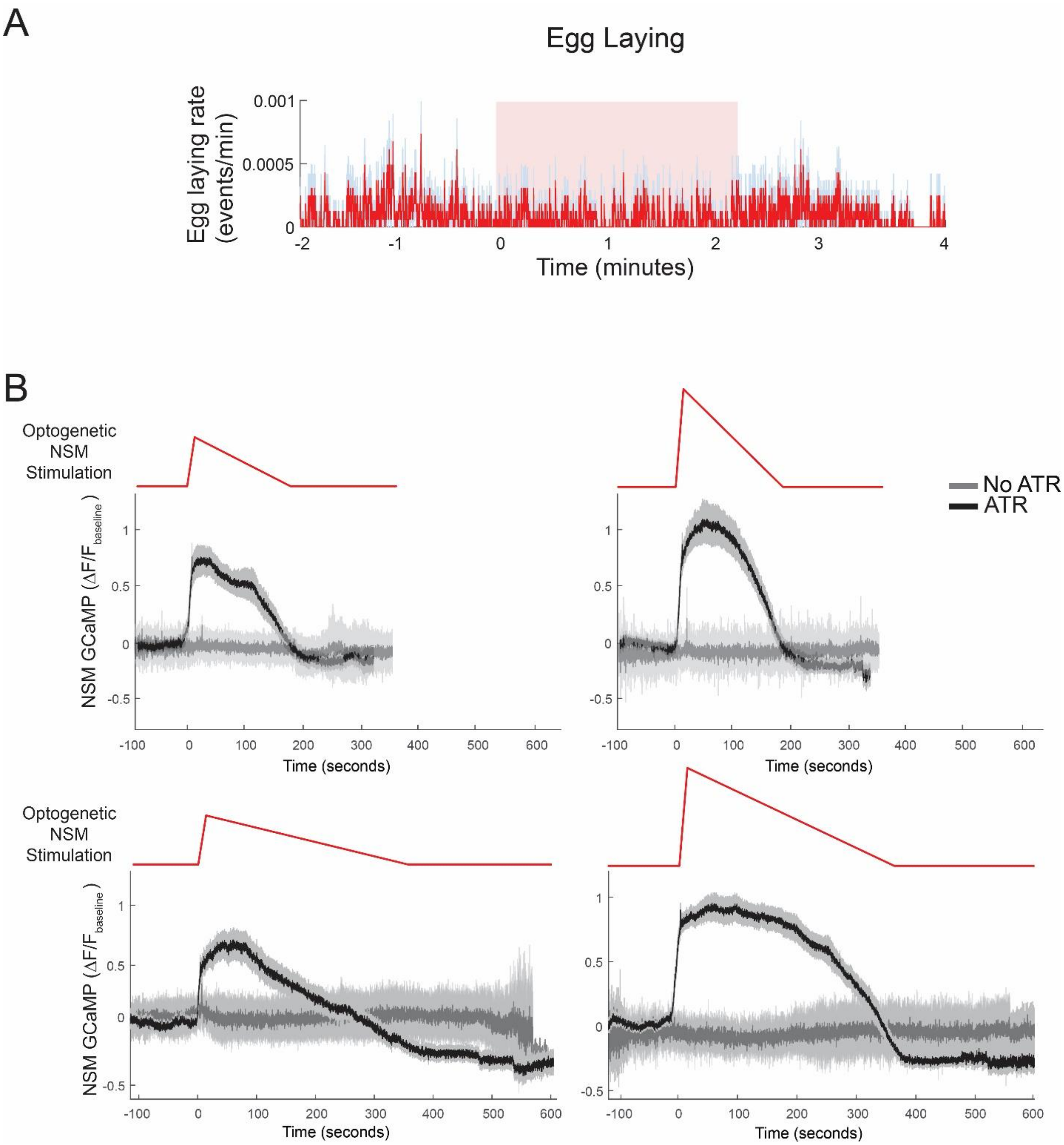

Figure S1, Related to Figure 1.

(A) Egg-laying behavior of animals in response to NSM::Chrimson activation. Note that NSM::Chrimson activation has no effect on egg-laying rates. Because of the lack of effect, this was not quantified in the serotonin receptor mutant strains. N=45 stimulation events (3 per animal).

(B) NSM GCaMP6m recordings during simultaneous NSM::Chrimson activation. Animals were immobilized with tetramisole and imaged under dim blue light conditions (with short light pulses) to prevent blue light activation of Chrimson. Red light was applied in the indicated temporal patterns to activate NSM::Chrimson. Note that brighter red light led to stronger NSM GCaMP

bioRxiv preprint doi: https://doi.org/10.1101/2023.01.15.524132; this version posted January 18, 2023. The copyright holder for this preprint (which was not certified by peer review) is the author/funder, who has granted bioRxiv a license to display the preprint in perpetuity. It is made available under aCC-BY 4.0 International license.

responses and more prolonged red light exposure led to longer NSM GCaMP activity bouts. N=27-59 animals per condition for +ATR conditions; n=10-23 animals for No-ATR conditions.

bioRxiv preprint doi: https://doi.org/10.1101/2023.01.15.524132; this version posted January 18, 2023. The copyright holder for this preprint (which was not certified by peer review) is the author/funder, who has granted bioRxiv a license to display the preprint in perpetuity. It is made available under aCC-BY 4.0 International license.

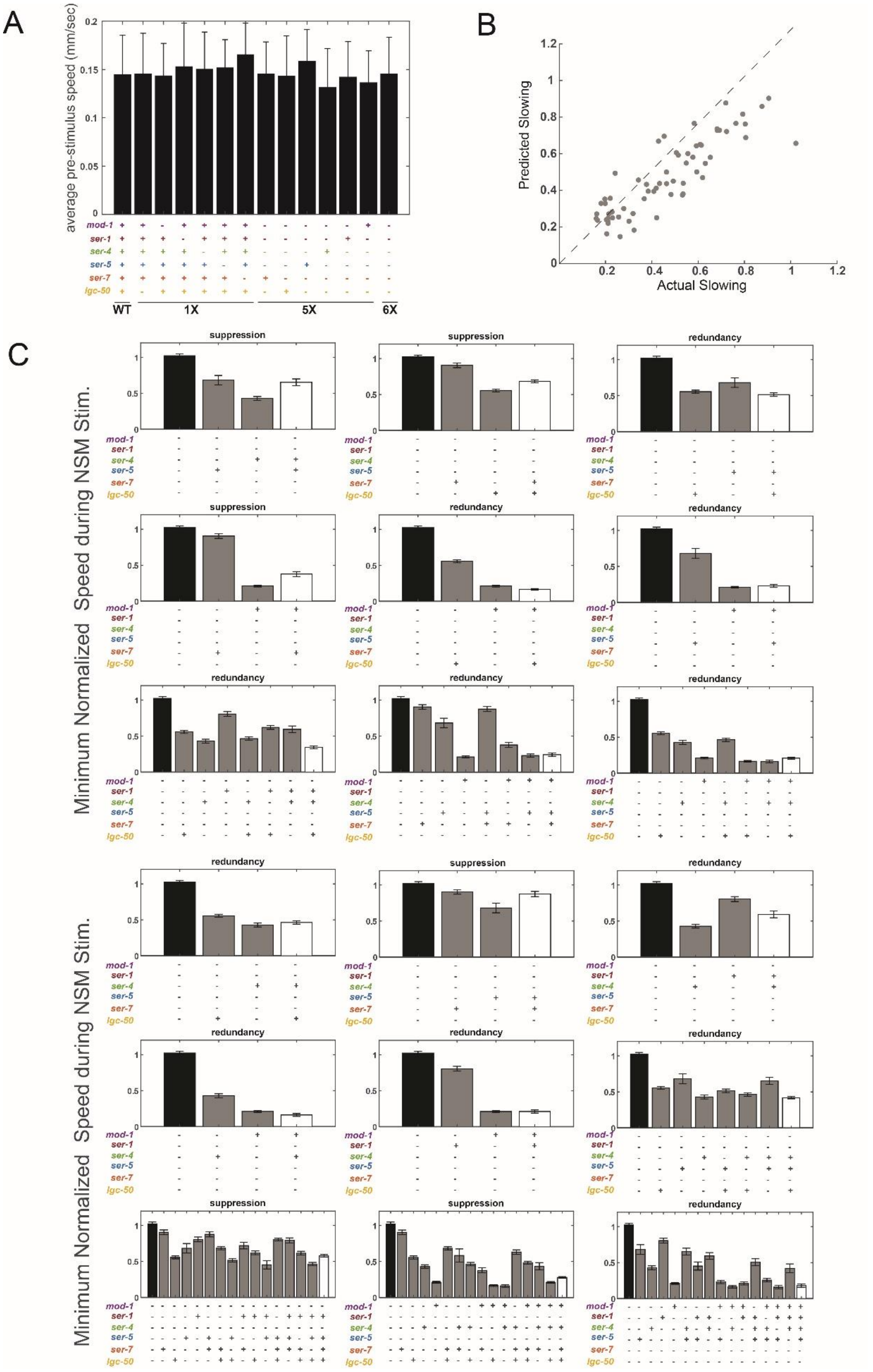

Figure S2, Related to Figure 2.
(A) Baseline speeds of animals of the indicated genotypes prior to optogenetic NSM stimulation. Note that baseline speeds are not significantly different from one another. n=45-496 animals per genotype. Data are shown as means ± standard deviation.

bioRxiv preprint doi: https://doi.org/10.1101/2023.01.15.524132; this version posted January 18, 2023. The copyright holder for this preprint (which was not certified by peer review) is the author/funder, who has granted bioRxiv a license to display the preprint in perpetuity. It is made available under aCC-BY 4.0 International license.

(B) Performance of the linear model (with interaction terms) that predicts NSM-induced slowing, using a train/test design where the model was trained on data from 63 of the 64 serotonin receptor mutant genotypes and then used to predict the slowing behavior of the withheld genotype. This procedure was repeated 64 times (once for each genotype) and the corresponding dots in the scatter plot are from these trials.

(C) Each plot here depicts a set of mutant phenotypes related to a single interaction term that was added to the linear model that predicts NSM-induced slowing behavior based on animals' genotypes. The statistical criteria to add an interaction term to the model is described in Methods. Here, to make clear what these interaction terms capture about receptor interactions, we display a series of slowing phenotypes in each panel. For each interaction term, we display the NSM-induced slowing behavior of the compound mutant that corresponds to the interaction term. For example, there was an interaction term in the linear model that allows the presence of *ser-5* to inhibit slowing when *ser-4* is present (top left). For this interaction term, we display the slowing behavior of 6X 5-HTR animals, animals with only *ser-4* or *ser-5* present, and animals with both *ser-4* and *ser-5* present. The plot labels indicate the type of interaction between the receptors: suppression or redundancy (no examples of synergistic interactions were detected).

bioRxiv preprint doi: https://doi.org/10.1101/2023.01.15.524132; this version posted January 18, 2023. The copyright holder for this preprint (which was not certified by peer review) is the author/funder, who has granted bioRxiv a license to display the preprint in perpetuity. It is made available under aCC-BY 4.0 International license.

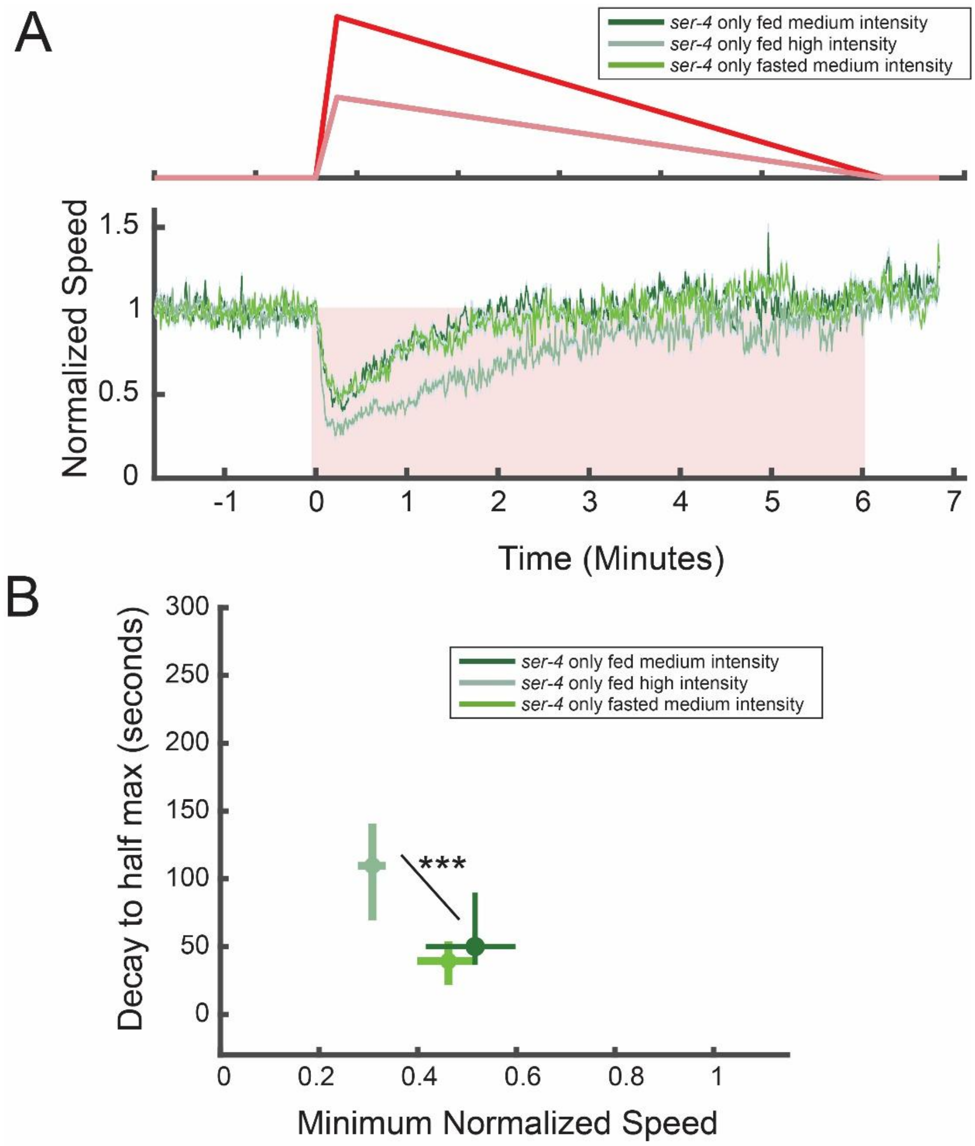

Figure S3, Related to Figure 3.

(A) Changes in animal speed for *ser-4*-only mutant animals in response to NSM::Chrimson activation with the indicated waveforms of light. Note that data are from fed or fasted animals, as indicated. Compared to medium-amplitude NSM::Chrimson stimulation in fed animals, fasted animals show no enhancement in slowing. However, stronger-amplitude NSM::Chrimson stimulation in fed animals does lead to a larger change in speed, compared to medium-amplitude stimulation in fed animals. Thus, fasting animals does not exaggerate slowing in *ser-4*-only mutants and this is not due to a saturation effect. Data are quantified in panel (B)

(B) Quantification of data in (A). Data are shown as means ± 95% confidence interval. N=83-103 animals per condition. ***p<0.001, empirical p-value that difference between slowing distributions is non-zero.

bioRxiv preprint doi: https://doi.org/10.1101/2023.01.15.524132; this version posted January 18, 2023. The copyright holder for this preprint (which was not certified by peer review) is the author/funder, who has granted bioRxiv a license to display the preprint in perpetuity. It is made available under aCC-BY 4.0 International license.

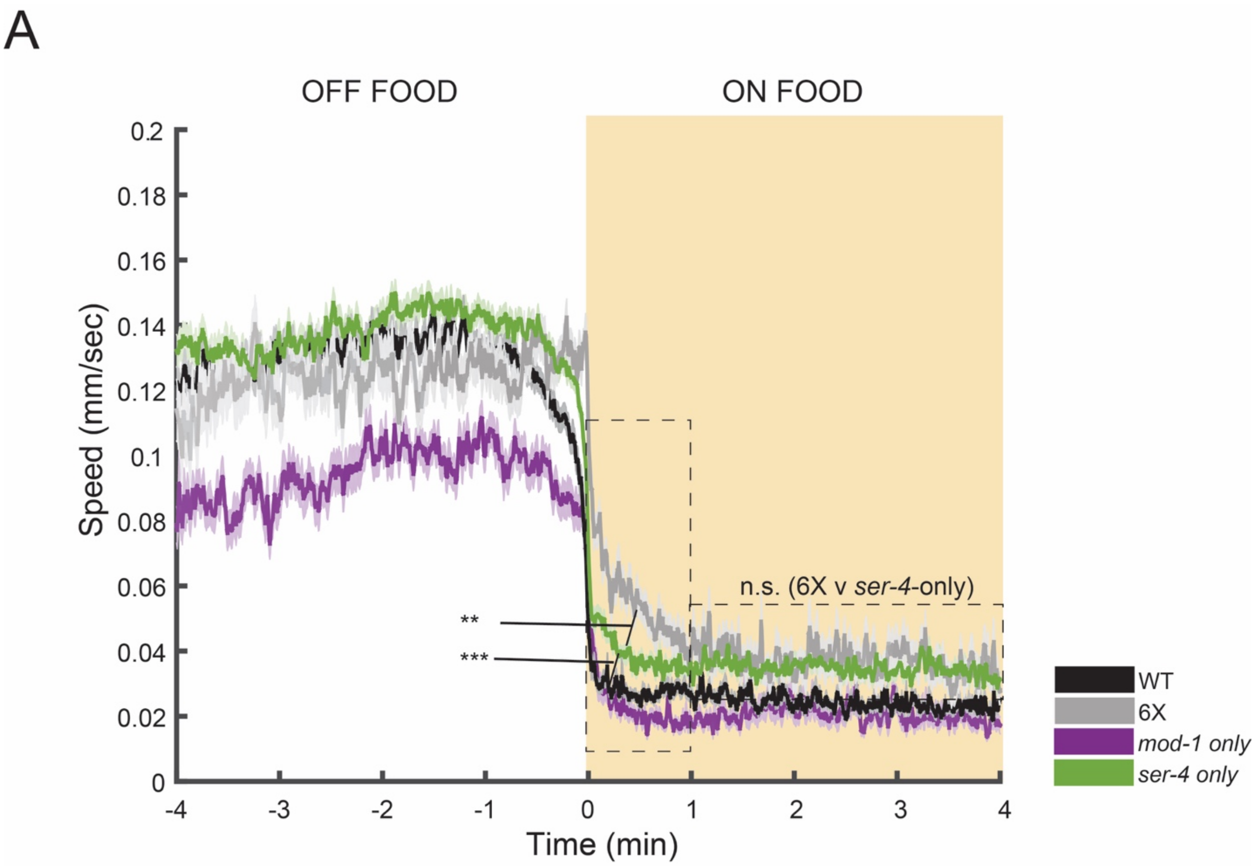

Figure S4, Related to Figure 4

(A) Animal speed for the indicated genotypes shown as event triggered averages surrounding bacterial food patch encounters. Data are shown as means ± SEM. N= 81-221, **p<0.01, ***p<0.001, two-sided Wilcoxon rank sum test between the indicated groups over the indicated time ranges.

bioRxiv preprint doi: https://doi.org/10.1101/2023.01.15.524132; this version posted January 18, 2023. The copyright holder for this preprint (which was not certified by peer review) is the author/funder, who has granted bioRxiv a license to display the preprint in perpetuity. It is made available under aCC-BY 4.0 International license.

A

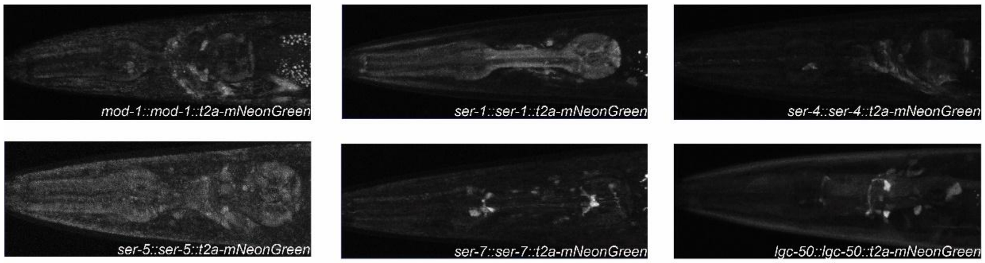

B

mod-1

Neurons	Fed	Starved
AVE	100%	100%
RME	100%	100%
RMD	100%	100%
PVN	78%	100%
ADE	88%	100%
AIY	100%	100%
AIZ	100%	100%
NSM	100%	100%
RIS	100%	100%
PVC	78%	100%
AIM	100%	100%
ALA	100%	100%
RIC	100%	100%
RID	100%	100%
RIF	100%	100%
ALN	100%	100%
DVB	100%	71%
	n=9	n=7

ser-1

Neurons	Fed	Starved
AVE	100%	100%
IL1	100%	100%
RME	100%	100%
RMD	100%	100%
PVN	100%	100%
URX	100%	100%
AIB	90%	100%
DVC	80%	100%
I1	100%	100%
I2	100%	100%
I3	100%	100%
IL2	100%	100%
PVQ	90%	100%
PVT	100%	100%
RIA	100%	100%
URY	100%	100%
	n=10	n=8

ser-4

Neurons	Fed	Starved
AVE	100%	100%
IL1	100%	100%
RME	85%	100%
RMD	100%	100%
PVN	100%	100%
ADE	100%	0%
AIY	100%	100%
AIZ	100%	100%
NSM	100%	100%
RIS	100%	100%
PVC	100%	100%
URX	100%	100%
AIN	100%	50%
AQR	100%	100%
AVA	100%	100%
BAG	100%	100%
DVA	85%	16%
M1	100%	100%
PVR	100%	100%
RIM	100%	100%
RIV	100%	100%
SAA	85%	100%
SMD	100%	100%
VB2	100%	100%
	n=7	n=6

ser-5

Neurons	Fed	Starved
AVE	85%	0%
IL1	85%	0%
RME	71%	100%
RMD	57%	78%
ADE	100%	100%
AIY	100%	100%
AIZ	100%	100%
NSM	100%	100%
RIS	100%	100%
AIM	100%	100%
ALA	100%	100%
RIC	100%	100%
RID	100%	100%
RIF	100%	100%
	n=7	n=9

ser-7

Neurons	Fed	Starved
IL1	100%	100%
I2	100%	100%
ASJ	100%	100%
CEP	100%	100%
I5	100%	100%
I6	100%	100%
M3	100%	100%
M4	100%	100%
M5	100%	100%
	n=5	n=7

lgc-50

Neurons	Fed	Starved
I2	100%	100%
M3	100%	100%
RME	85%	100%
RMD	100%	100%
DVB	85%	0%
URY	100%	100%
BAG	85%	66%
RIM	100%	100%
SMD	71%	100%
AIA	100%	100%
AUA	100%	0%
DD1	85%	100%
PHA	100%	100%
	n=7	n=6

0% 25% 50% 75% 100%

Figure S5, Related to Figure 5.

(A) Images of the fluorescent reporters for the six serotonin receptor genes, shown without overlaying NeuroPAL images. Details about construction of the fluorescent reporters are in Fig. 5A.

bioRxiv preprint doi: https://doi.org/10.1101/2023.01.15.524132; this version posted January 18, 2023. The copyright holder for this preprint (which was not certified by peer review) is the author/funder, who has granted bioRxiv a license to display the preprint in perpetuity. It is made available under aCC-BY 4.0 International license.

(B) Results of cell identification from the six strains expressing t2a-mNeonGreen reporters from each serotonin receptor gene. For each reporter line, we show the fraction of animals where each mNeonGreen was detected in each cell. Cells that are not listed had 0% detected.

bioRxiv preprint doi: https://doi.org/10.1101/2023.01.15.524132; this version posted January 18, 2023. The copyright holder for this preprint (which was not certified by peer review) is the author/funder, who has granted bioRxiv a license to display the preprint in perpetuity. It is made available under aCC-BY 4.0 International license.

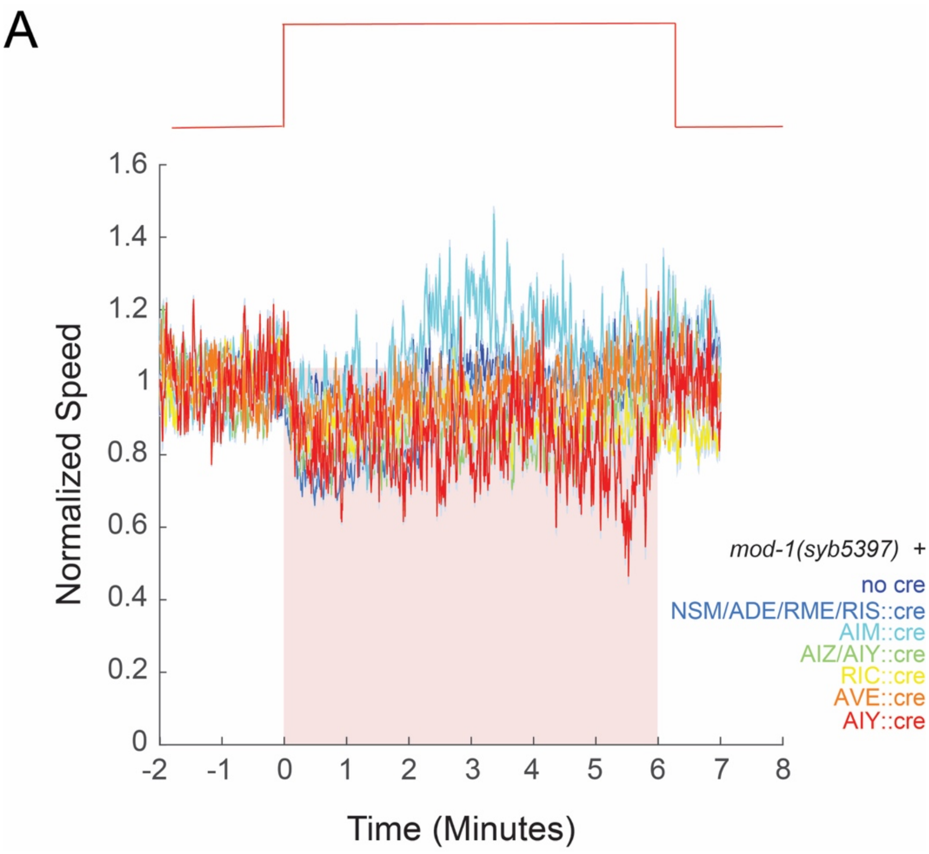

Figure S6, Related to Figure 6.

(A) Changes in animal speed in response to NSM::Chrimson stimulation in the indicated *mod-1* rescue strains. Note that genetic rescues in these indicated neurons did not lead to a rescue of NSM-induced slowing, compared to the mod-1 inverted allele. N=29-116 animals per condition. Promoters used for Cre expression were: *Pcat-1* (NSM, ADE), *Punc-25* (RME, RIS), *Pser-2b* (AIY, AIZ), *Ptbh-1* (RIC), *Popt-3* (AVE), *Pnlp-70* (AIM), *Pttx-3* (AIY).

bioRxiv preprint doi: https://doi.org/10.1101/2023.01.15.524132; this version posted January 18, 2023. The copyright holder for this preprint (which was not certified by peer review) is the author/funder, who has granted bioRxiv a license to display the preprint in perpetuity. It is made available under aCC-BY 4.0 International license.

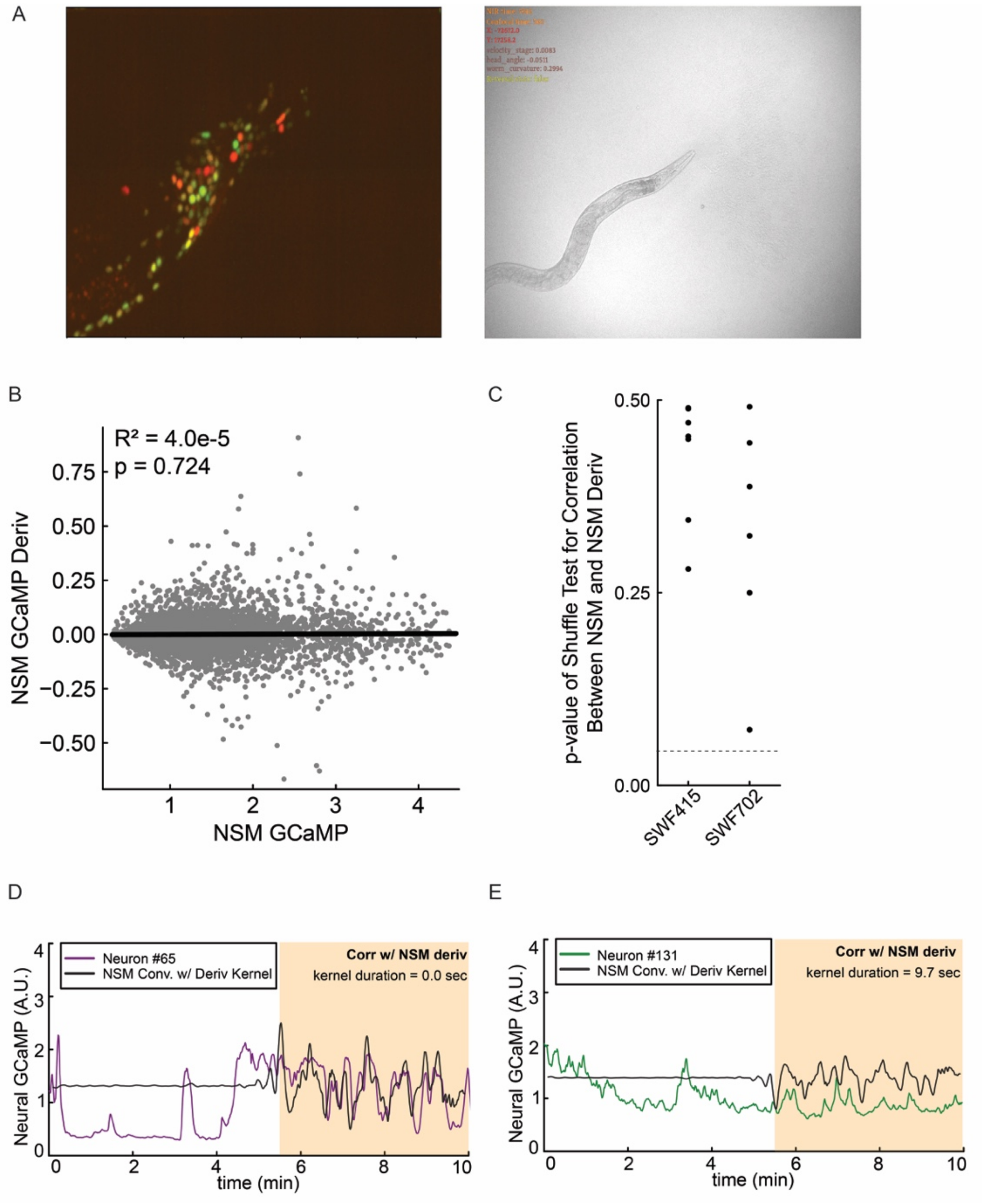

Figure S7, Related to Figure 7.

(A) Example images from a brain-wide recording. Left: A maximum intensity project of a single timepoint, showing the NLS-GCaMP7f (green) and NLS-mNeptune2.5 (red) signals in the head. Right: Behavioral image, captured in near infrared brightfield.

(B) Scatterplot showing NSM activity and its derivative. Each dot is a timepoint and multiple animals are pooled together here. There is no significant correlation between the two.

(C) Results of a statistical test to examine whether NSM's activity was correlated with NSM derivative. This is related to panel B, except here we are performing the exact statistical test used throughout Figures 7 and 8 to test whether NSM itself is significantly correlated with its derivative (i.e. NSM activity convolved with filters that take its derivative). For all the animals recorded in Figure 7 (SWF415) and in Figure 8 (SWF702), there was no correlation.

bioRxiv preprint doi: https://doi.org/10.1101/2023.01.15.524132; this version posted January 18, 2023. The copyright holder for this preprint (which was not certified by peer review) is the author/funder, who has granted bioRxiv a license to display the preprint in perpetuity. It is made available under aCC-BY 4.0 International license.

(D) Two example neurons that are also displayed in Fig. 7G that showed a significant positive (left) or negative (right) correlation with NSM's derivative. Here, we show these neural traces again, but they are overlaid with NSM that has been convolved with a differentiator kernel (i.e. showing NSM derivative) so that this relationship can be more easily inspected. Note that the differentiator kernel on the right has a flipped sign, so that the black trace is basically the inverse of the derivative of NSM.

bioRxiv preprint doi: https://doi.org/10.1101/2023.01.15.524132; this version posted January 18, 2023. The copyright holder for this preprint (which was not certified by peer review) is the author/funder, who has granted bioRxiv a license to display the preprint in perpetuity. It is made available under aCC-BY 4.0 International license.

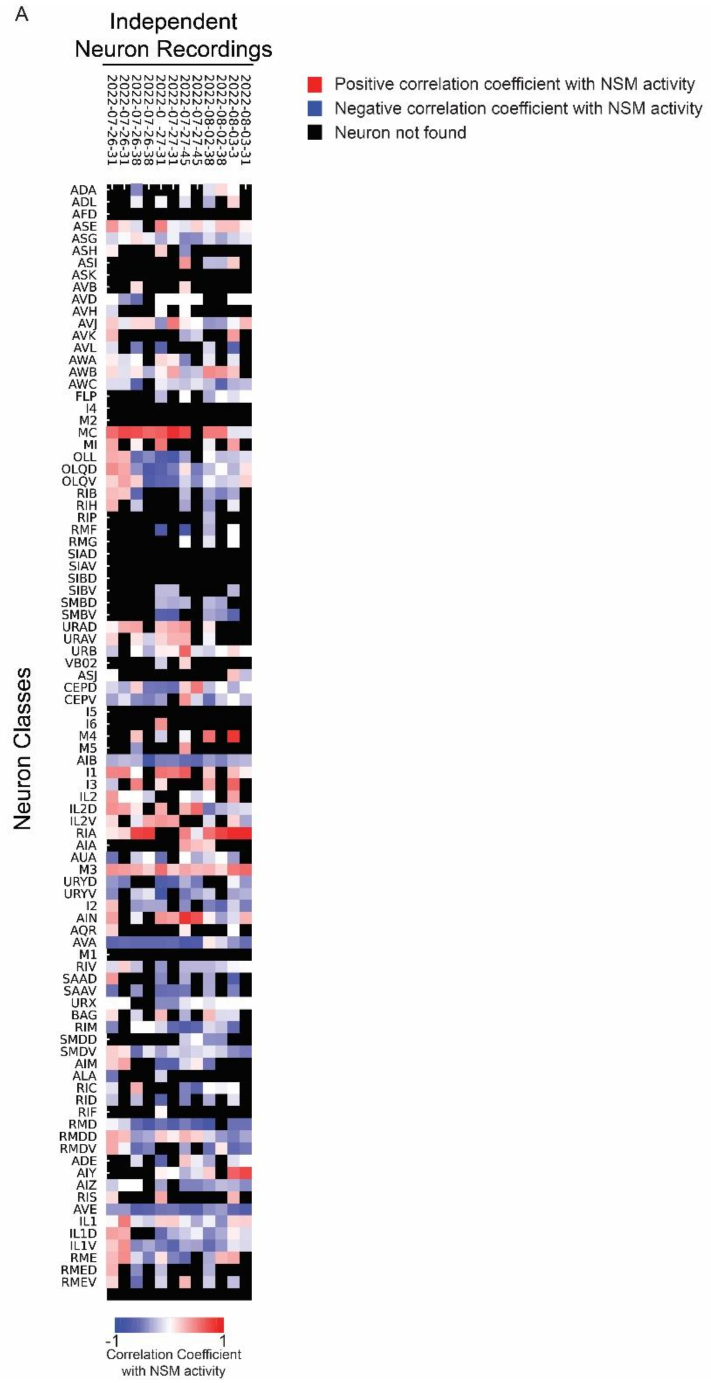

1822

1823 **Figure S8, Related to Figure 8**

1824 (A) Heat map depicting how each neuron class' activity is associated with NSM activity in each
1825 recording. Neuron classes are shown as rows and the columns are the different recordings
1826 of that neuron (left and right neurons are shown separately). Pixels are black if the neuron
1827 was not found (or if the neuron class exists as a single cell).
